# In vitro immunomonitoring of insect venom-allergic patients on immunotherapy

**DOI:** 10.1186/2045-7022-4-S2-O3

**Published:** 2014-03-17

**Authors:** Liliana Cifuentes, Mathias Schnedler, Simon Blank, Davide Pennino, Markus Ollert, Lukas Balzer, Henning Seismann, Ulf Darsow, Edzard Spillner, Johannes Ring

**Affiliations:** 1 Department of Dermatology and Allergy, Biederstein, Technical University Munich, Biederseiner Str. 29, Munich, 80802, Germany; 2 Technical University Munich, Department of Dermatology and Allergy, Biederstein, Munich, Germany; 3 University of Hamburg, Institute of Biochemistry and Molecular Biology, Hamburg, Germany; 4 Technische Universität and Helmholtz Center Munich, ZAUM – Center of Allergy and Environment (ZAUM), Munich, Germany

## Objective

Sting challenge is the gold standard method to evaluate the therapeutic efficiency of allergen specific immunotherapy (ASIT) in hymenoptera venom allergic patients. Unfortunately, this method is risky, expensive and time consuming. Therefore, the development of an in vitro method is desirable. Recently the basophil activation test (BAT) performed with natural venom has been shown to be a promising method. We aimed to improve the benefit of BAT technology by applying the panel of recombinant allergens Ves v 1, Ves v 2, Ves v 3 and Ves v 5.

## Methods

BAT was performed in 83 patients with hymenoptera venom allergy. Patients were evaluated before and one year after starting SIT before the sting challenge.

## Results

Natural venom and Ves v 5 recognise the majority of wasp venom allergic patients. The BAT reactivity towards natural venom and recombinant Ves v 5 is diminished during ASIT. While the majority of patients without allergic systemic reaction after the sting challenge did not induce basophil activation towards natural venom, patients with an allergic reaction after the sting challenge were positive to BAT towards natural venom and Ves v 5. BAT performed with natural venom and recombinant allergens is a promising in vitro method to predict successful immunotherapy and new allergen sensitization of patients upon allergen immuntherapy

